# Benazepril hydrochloride improves diabetic nephropathy and decreases proteinuria by decreasing ANGPTL-4 expression

**DOI:** 10.1186/s12882-017-0724-1

**Published:** 2017-10-04

**Authors:** Lingyu Xue, Xiaoqing Feng, Chuanhai Wang, Xuebin Zhang, Wenqiang Sun, Kebo Yu

**Affiliations:** Department of Nephrology, the Affiliated Hospital of Taishan Medical University, No. 706 Taishan Street, Taian, Shandong Province 271000 China

**Keywords:** Benazepril hydrochloride, Diabetic nephropathy, ANGPTL-4, Proteinuria

## Abstract

**Background:**

This study aimed to investigate the effects of benazepril hydrochloride (BH) on proteinuria and ANGPTL-4 expression in a diabetic nephropathy (DN) rat model.

**Methods:**

A total of 72 Wistar male rats were randomly divided into three groups: normal control (NC), DN group and BH treatment (BH) groups. The DN model was induced by streptozotocin (STZ). Weight, glucose, proteinuria, biochemical indicators and the kidney weight index were examined at 8, 12 and 16 weeks. In addition, ANGPTL-4 protein and mRNA expressions were assessed by immunohistochemistry and qRT-PCR, respectively. Relationships between ANGPTL-4 and biochemical indicators were investigated using Spearman analysis.

**Results:**

Weight was significantly lower but glucose levels were significantly higher in both the DN and BH groups than in the NC group (*P* < 0.05). Compared with the DN group, proteinuria, urea, creatinine, triglycerides and total cholesterol levels were decreased, whereas the albumin level was increased after BH treatment (all *P* < 0.05). Furthermore, BH diminished kidney volume and ameliorated the pathological changes associated with DN. ANGPTL-4 expression was significantly decreased after BH treatment, and ANGPTL-4 expression was highly correlated with biochemical indicators of DN (*P* < 0.05).

**Conclusions:**

Benazepril hydrochloride improves DN and decreases proteinuria by decreasing ANGPTL-4 expression.

## Background

Diabetic nephropathy (DN) is one of the major chronic microvascular complications of diabetes, which seriously impacts a large number of people worldwide [1, 2]. DN incidence is increasing with the rapid growth of diabetes year by year, and DN has become a major cause of end-stage renal disease (ESRD) [3]. According to the statistics of the International Diabetes Federation (IDF), the number of diabetic patients has reached 37 million worldwide, with about 4.6 million of them dying in 2011. Furthermore, rapid growth in the incidence of diabetes is occurring in China and other developing countries, leading to a heavy social and economic burden. DN has become the first major disease requiring dialysis treatment, with expensive associated costs [4].

Microalbuminuria is generally the first clinical manifestation of DN, and it further promotes clinical progression. Without effective intervention, proteinuria levels further increase and eventually leads to kidney failure. Proteinuria is not only a marker for DN severity but is also closely associated with DN progression.

A recent study has found that vascular endothelial dysfunction is important for diabetic vascular disease progression [5]. Multiple angiopoietin-related genes, such as Ang1, Ang2, ANGPTL-1, ANGPTL-2 and ANGPTL-3, are critical for angiogenesis [6, 7]. ANGPTL-4 is homologous with Ang2 at the amino acid level, suggesting that ANGPTL-4 may be associated with angiogenesis. ANGPTL-4 overexpression in rats induce and increase nephrotic-range proteinuria, whereas proteinuria is markedly lower in ANGPTL-4 knock-in mice [8]. Increasing evidence has shown that ANGPTL-4 is overexpressed in minimal change nephropathy, which is correlated with proteinuria [9, 10]. Benazepril hydrochloride (BH) is a commonly used drug for the clinical treatment of DN, which not only decreases blood pressure but also decreases the proteinuria level [11]. Although several studies have investigated how BH improves DN, the underlying mechanism is still far from completely clear and the effect of DN on ANGPTL-4 remains to be elucidated.

In the present study, we aimed to investigate the relationship between ANGPTL-4 and biochemical indices as well as proteinuria. The effects of BH on biochemical indices, proteinuria and ANGPTL-4 expression were also assessed. Our study provides further information on the molecular mechanism of BH and supplies new insights for drug development of DN by targeting ANGPTL-4.

## Methods

### Ethics statement

Animals were treated strictly in accordance with all animal care and experimental procedures according to the Care and Use of Laboratory Animals. All animal experiments were permitted and approved by the Committee of our hospital and all efforts were made to minimise discomfort and pain of rats.

### Animals and grouping

A total of 90 healthy Wistar male rats, aged 6–8 weeks, weighing 170–200 g, were purchased from Jining Lukang Pharmaceutical Co., Ltd. (Shandong, China). All the rats were housed in a specific pathogen-free animal room at a temperature of 25 °C ± 2 °C, a relative humidity of 25%–30% and a standard 12-h light/dark cycle. The rats had free access to sterilised water and food. After an adaptive feeding period of 7 days, blood glucose test strips were applied to screen out ineligible rats with a fasting plasma glucose of >7.0 mmol/L and a urine protein score ≥ 1. Seventy-two eligible rats were randomly assigned into three groups according to a random number table, namely a normal control group (NC group, *n* = 24), a DN group, n = 24) and a BH-treated group (BH group, n = 24). At the same time, 12 additional rats were randomly divided into DN and BH groups for a synchronisation test to avoid excess mortality in the experiments, which might influence the statistical outcome.

### DN rat model

Rats were fasted for 12 h before diabetes was induced in DN and BH rats with an intraperitoneal injection of 1% streptozotocin (STZ) at a dose of 60 mg/kg body weight. The rats in the NC group received an equal volume of saline. Blood from the tail vein was used to detect plasma glucose by an ACCU-CHEK blood glucose monitoring device (Roche) after 3 days. Development of the diabetes model was successful if the plasma glucose was >16.7 mmol/L. Three weeks later, urinary protein levels in urine collected over 24 h were assessed for each rat. Urinary protein ≥30 mg/24 h indicated successful development of DN in the rat model. The rats in the BH group were then gavage fed BH (Lotensin, Beijing Novartis Pharma Ltd., Beijing, China) at a dose of 10 mg/kg/d, and the rats in the NC and DN groups were fed with an equal volume of saline. Rats were closely monitored during the whole experiment, including weight, diet, drinking, spirit and colour, etc.

### Sample collection and serum parameter measurements

Blood samples were obtained by tail venepuncture to examine the glucose levels. Blood samples taken from the heart of anaesthetised rats were immediately analysed to measure serum levels of urea, creatinine, triglycerides, total cholesterol and albumin using an automatic biochemistry analyser (AU5800, Beckman Coulter Inc., Brea CA, USA). Urine was collected for 1 day before sacrifice to detect proteinuria using the nephelometry method (Siemens BN II, Deerfield, IL, USA).

### Histological observation

At weeks 4, 8 and 12 after BH induction, eight rats from each group were sacrificed under anaesthesia to obtain the kidneys, which were fixed with 10% formalin for 24 h and dehydrated with graded ethanol. After transparency, the specimens were embedded into paraffin and cut into 3-μm sections. The sections were then dewaxed, rehydrated and stained with haematoxylin and eosin (H&E) for observation under an inverted microscope (Nikon YS100, Tokyo, Japan).

### Immunohistochemistry

After dewaxing, rehydration and antigen retrieval, the sections were blocked with goat serum for 10–15 min, followed by incubation with specific primary antibody at 37 °C for 2 h. Then the sections were washed with phosphate-buffered saline (PBS) three times and incubated with biotin-labelled goat anti-rabbit IgG at 37 °C for 15 min. Subsequently, the sections were incubated with horseradish peroxidase (HRP)-labelled streptavidin, stained with 3,3′-diaminobenzidine and re-stained with haematoxylin. A microscope (Olympus, Japan) was used to observe staining and five random images were taken for each section. In addition, Image-Pro Plus software 6.0 (Media Cybernetics, Silver Spring, MD, USA) was used for image analysis, and the average optical density value was calculated to reflect the expression level of the proteins.

### Quantitative real-time PCR

Rat kidney samples (~100 mg) were used for extraction of total RNA by TRIzol reagent (Tiangen, Beijing, China). A reverse transcription kit (Tiangen) was used to obtain cDNA. The primer sequences for real-time PCR were as follows: Angptl-4 primers 5′-TCT CAC TTC TCG CCT ACC AG-3′ and 5′-CCC TAT CTC CAG TCG GTC AA-3′, GAPDH primers 5′-TTC TAG AGA CAG CCG CAT CT-3′ and 5′-TGG TAA CCA GGT GTC CGA TA-3′. For real-time PCR, a 2× SYBR Green PCR Master Mix kit (Applied Biosystems, Warrington, UK) was used according to the manufacturer’s instructions, and amplified with a BioRad machine (Singapore). Amplification conditions were set as 94 °C for 15 min; 40 cycles of 94 °C for 20 s, 58 °C for 30 s, 68 °C for 30 s; then 68 °C for 10 min. The mRNA level of Angptl-4 was normalised to GAPDH and the results were analysed using the 2^−△△Ct^ method.

### Statistical analysis

All data were expressed as mean ± standard deviation (SD) and analysed using SPSS 19.0. The difference comparisons were performed by one-way analysis of variance followed by multiple comparisons with the LSD test. Correlation analysis between two variables was conducted using Spearman analysis. *P* < 0.05 was considered as a statistically significant difference.

## Results

### Basic characteristics of rats in the different groups

In the NC group, rats showed normal behaviour, a shiny coat, produced normal amounts of urine without a peculiar smell, with no diarrhoea or death. Rats in the DN group showed signs of distress with less activity, body weight loss, dark and brown hair, an increased water and food intake and a larger urine volume with a peculiar smell. In addition, some rats had ring ulceration, eye lesions and diarrhoea. After treatment with BH, the basic characteristics of diabetic rats were notably improved. During the entire experiment, the number of dead rats was zero, five and two in the NC, DN and BH groups, respectively. The weights of the rats are shown in Table 1. Compared with the NC group, the weights were significantly decreased in both the DN and BH groups throughout the whole experiment (*P* < 0.05). Significant differences were also found between the DN and BH groups at 16 weeks (*P* < 0.05), but not at 8 and 12 weeks (*P* > 0.05). The weight of rats in the NC group kept increasing (*P* < 0.05), whereas no obvious changes were found in the DN and BH groups (*P* > 0.05).

**Table 1:** The weights (g) of rats in the different experimental groups (n = 8 for each group at each time point)

Time (weeks)	NC group	DN group	BH group
8	375.90 ± 25.072	194.30 ± 13.684 ^*^	212.98 ± 15.187 ^*#^
12	503.33 ± 27.830	181.33 ± 12.055 ^*^	224.78 ± 13.826 ^*#^
16	538.50 ± 28.116	187.00 ± 12.247 ^*^	269.93 ± 18.291 ^*#^

*NC* normal controls, *DN* diabetic nephropathy, *BH* benazepril treated group

The difference comparisons were performed by one-way analysis of variance followed by multiple comparisons with the LSD test. *P < 0.05 vs. NC, ^#^ *P* < 0.05 vs. DN

### Changes of biochemical indicators

**Fig. 1:**
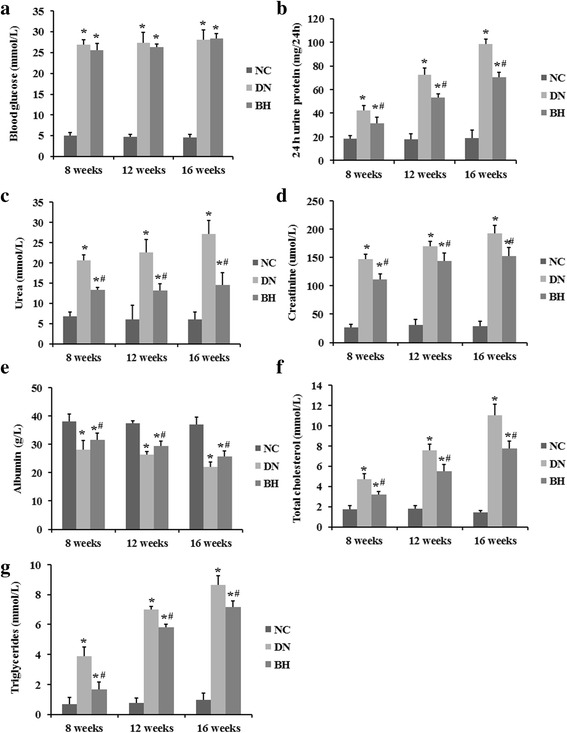
Changes of biochemical indicators in the different groups. **a** Blood glucose; **b** 24-h urinary protein; **c** Urea; **d** Creatinine; **e** Albumin; **f** Total cholesterol; **g** Triglycerides. NC: normal controls; DN: diabetic nephropathy; BH: benazepril hydrochloride treatment group; *P < 0.05 vs. NC, ^#^ *P* < 0.05 vs. DN. (*n* = 8 for each group at each time point)

Glucose, proteinuria, urea, creatinine, triglycerides, total cholesterol and albumin levels are shown in Fig. 1. The results show that after treatment with STZ, the glucose levels in both the DN (26.910 ± 1.115) and BH (25.611 ± 1.587) groups were >16.7 mmol/L at week 8, and the level of 24-h proteinuria in the DN (42.525 ± 4.157) and BH (31.281 ± 5.123) groups were both >30 mg/24 h at week 8, which suggested that the rats were diabetic and that the DN rat models were successfully constructed. Glucose and proteinuria levels were significantly higher in the DN and BH groups than in the NC group (Fig. 1a and b, *P* < 0.05). After BH treatment, proteinuria levels significantly decreased compared with the DN group, although no significant difference in glucose levels was found between these two groups. In accordance with the marked reduction in proteinuria, urea, creatinine, triglycerides and total cholesterol levels were also decreased, whereas albumin levels were increased after BH treatment (Fig. 1c–g, *P* < 0.05).

### BH treatment diminished volume and the infiltration of inflammatory cells in DN rat kidneys

We used the kidney weight index (weight of unilateral kidney weight / body weight) to represent the increase in kidney volume (Table 2). As a result, increased kidney volume was found in the model (DN and BH) groups (*P* < 0.05) and BH treatment significantly decreased the kidney weight index compared with the DN group (*P* < 0.05).

**Table 2:** Kidney weight index (mg/g) in different groups (n = 8 for each group at each time point)

Time (weeks)	NC group	DN group	BH group
8	3.158 ± 0.505	7.059 ± 0.586^*^	5.894 ± 0.418^*#^
12	3.273 ± 0.520	8.024 ± 0.693^*^	6.103 ± 0.521^*#^
16	3.281 ± 0.299	9.211 ± 0.453^*^	6.723 ± 0.283^*#^

*NC* normal controls, *DN* diabetic nephropathy, *BH* benazepril treated group

The difference comparisons were performed by one-way analysis of variance followed by multiple comparisons with the LSD test. *P < 0.05 vs. NC, ^#^ *P* < 0.05 vs. DN

**Fig. 2:**
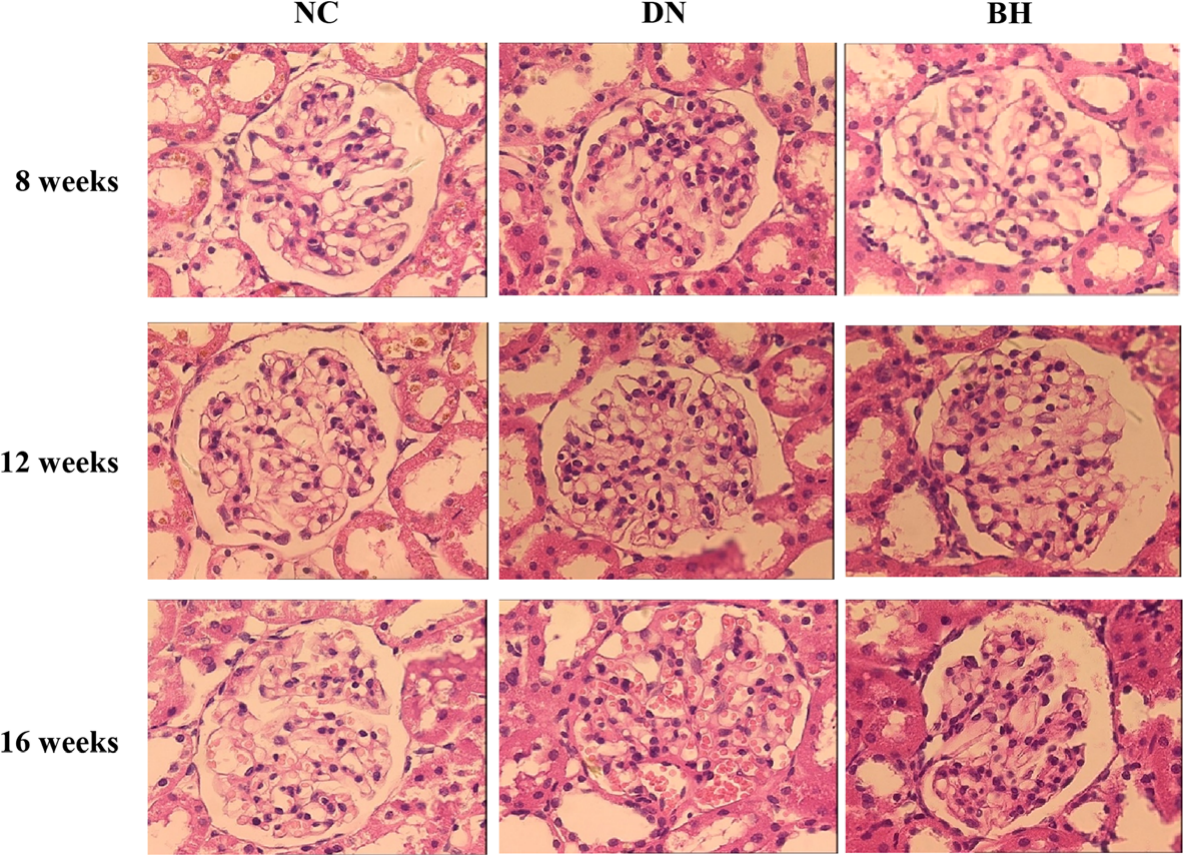
Benazepril hydrochloride improved the kidney histology in diabetic nephropathy rats. Histological observations with haematoxylin and eosin (H&E) staining (magnification, ×400); NC: normal controls; DN: diabetic nephropathy; BH: benazepril hydrochloride treatment group; *P < 0.05 vs. NC, ^#^ *P* < 0.05 vs. DN

Rats in the NC group had normal glomerular volume, shape, and structure (Fig. 2). For the DN group, the kidneys had bigger glomeruli, a thickened glomerular basement membrane, larger mesangial cells and a large amount of inflammatory cell infiltration. BH treatment markedly improved the pathological conditions, decreasing the mesangial cell size and matrix thickness, as well as dampening the infiltration of inflammatory cells (Fig. 2).

### BH treatment decreased ANGPTL-4 expression in DN rat kidneys

**Fig. 3:**
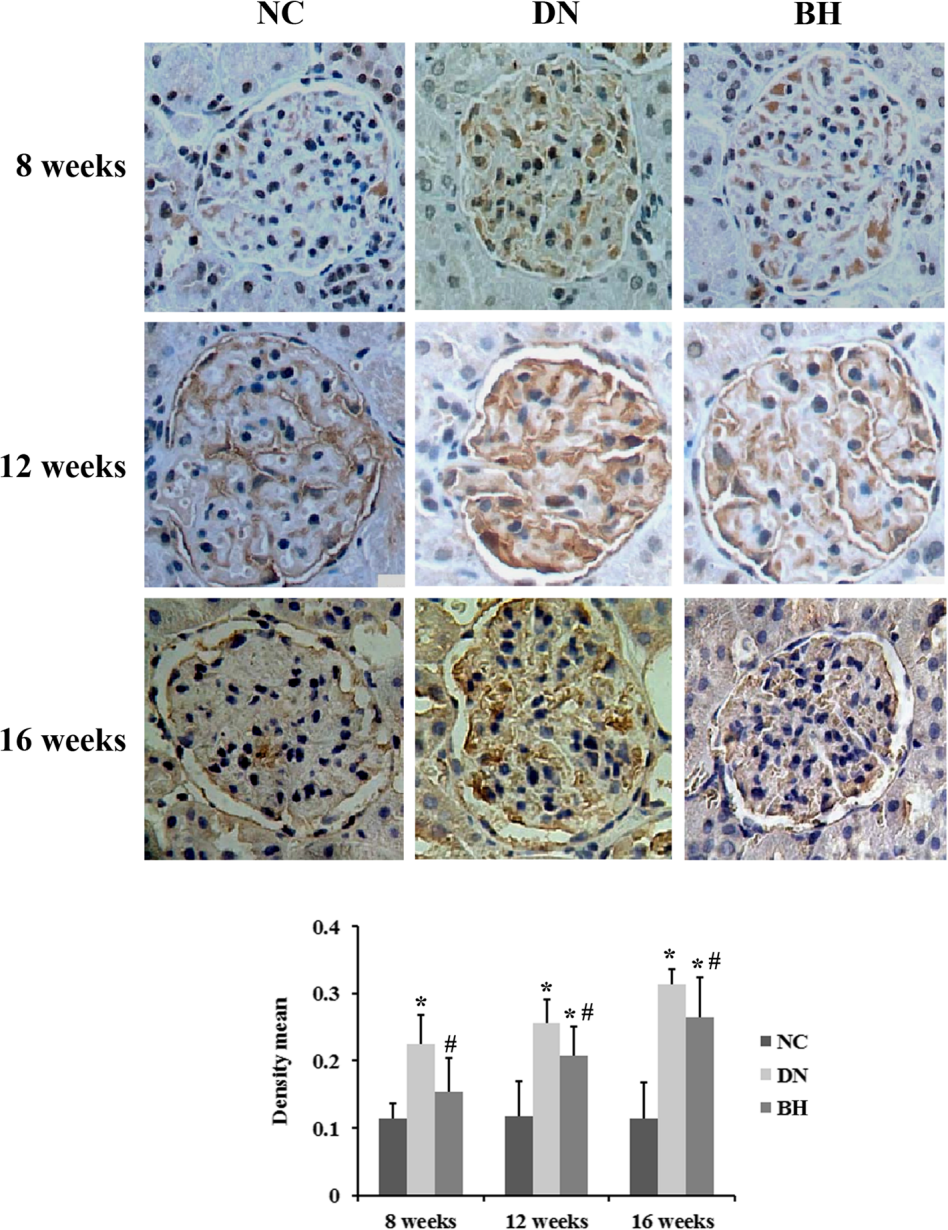
Benazepril hydrochloride decreased ANGPTL-4 expression in diabetic nephropathy rat kidneys. ANGPTL-4 protein levels in different groups examined by immunohistochemistry (magnification, ×400); NC: normal controls; DN: diabetic nephropathy; BH: benazepril hydrochloride treatment group; *P < 0.05 vs. NC, ^#^ *P* < 0.05 vs. DN (n = 8 for each group at each time point)

**Fig. 4:**
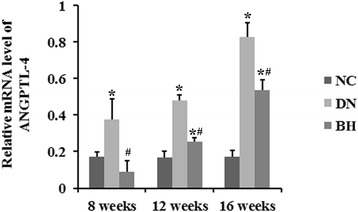
Benazepril hydrochloride decreased the mRNA level of ANGPTL-4 in diabetic nephropathy rat kidneys. NC: normal controls; DN: diabetic nephropathy; BH: benazepril hydrochloride treatment group; *P < 0.05 vs. NC, ^#^ *P* < 0.05 vs. DN (n = 8 for each group at each time point)

We noted that ANGPTL-4 expression was upregulated in the DN group at weeks 8, 12 and 16 compared with that in the NC group, as determined by immunohistochemistry (*P* < 0.05, Fig. 3). This finding was also confirmed at the mRNA level by qRT-PCR (Fig. 4). Further, an increase of ANGPTL-4 expression was found in the DN and BH groups at weeks 12 and 16 compared with that at week 8 (*P* < 0.05). Compared with the DN group, protein and mRNA levels of ANGPTL-4 were significantly decreased after treatment with BH (*P* < 0.05).

### The ANGPTL-4 level was significantly correlated with the biochemical indicators of DN

The correlations between ANGPTL-4 level and 24-h urine protein, total cholesterol, triglycerides, creatinine and kidney weight index were also analysed (Table 3). As a result, ANGPTL-4 levels positively correlated with the amount of 24-h urine protein, creatinine and the kidney weight index (*r* = 0.907, *P* < 0.01; *r* = 0.817, *P* < 0.01; *r* = 0.882, *P* < 0.01; respectively), whereas there was a negative correlation between the ANGPTL-4 level and total cholesterol (*r* = 0.536, *P* < 0.01).

**Table 3:** The correlations between ANGPTL-4 expression and biochemical indicators

		24-h urine protein	Albumin	Creatinine	Triglycerides	Total cholesterol	Kidney weight index
ANGPTL-4	r	0.907	−0.226	0.817	0.219	0.536	0.882
	p	0.000	0.160	0.000	0.174	0.000	0.000

Correlation analysis between two variables was conducted using Spearman analysis; r is the correlation coefficient and *P* < 0 05 was considered as significantly correlated

## Discussion

In this study, we showed that glomerular size and ANGPTL-4 expression were significantly increased, which most likely associated with the heavy proteinuria in DN rats. Furthermore, the ANGPTL-4 expression level significantly correlated with the amounts of 24-h urine protein and creatinine, the kidney weight index and total cholesterol levels. However, BH treatment markedly decreased ANGPTL-4 expression, which was accompanied by a reduction of proteinuria and an improved glomerular pathology in the DN rats.

An activated renin–angiotensin–aldosterone system is one of the most important contributors to DN pathogenesis [12]. Angiotensin-converting enzyme inhibitor (ACEI) drugs have been used for the treatment of STZ-induced diabetic rats and they decrease the expression of growth factor receptors and inhibit signalling [13]. Benazepril is a commonly used ACEI drug that has been used for the treatment of hypertension and heart failure [14, 15]. Recently, benazepril has been shown to generate a strong flow-mediated vasodilation response and a remarkable reduction in C-reactive protein in diabetic patients [16]. Meanwhile, BH has been widely used for renal diseases, such as renal hypertension, renal failure and DN [17, 18]. According to our results, BH notably improved the morphology of glomeruli and decreased the proteinuria in DN rats, suggesting that BH was an effective treatment for DN in the rat model.

ANGPTL-4 is involved in wound healing, cancer, angiogenesis and redox regulation, as well as lipid and glucose metabolism. Initial studies have revealed increased podocyte expression of ANGPTL-4 in minimal change disease (MCD), and it causes increased proteinuria in membranous nephropathy (MN) [8, 19]. Consistent with the previous studies, ANGPTL-4 was also upregulated in DN rats in our study. Downregulating ANGPTL-4 expression or promoting the conversion of ANGPTL-4 has been shown to decrease proteinuria in rats with MCD and DN [8, 20]. Considering ANGPTL-4 is a major molecular mediator of nephrotic syndrome, it has been identified as an ideal candidate for the treatment of proteinuric disorders caused by chronic kidney disease [20]. ManNAc decreases proteinuria in MCD rats, accompanied by a decrease of ANGPTL-4 expression [8]. Tacrolimus is a calcineurin inhibitor and it can decrease proteinuria in MN by targeting ANGPTL-4 [21]. In this study, ANGPTL-4 expression was also significantly lower after BH treatment compared with the DN model group, which indicates that ANGPTL-4 might be involved in the treatment effects of BH on DN.

An increasing number of reports have described the involvement of cytokines in the occurrence and progression of DN [22, 23], and the infiltration of inflammatory cells was also found in our H&E analyses. Multiple studies have suggested that ANGPTL-4 is involved in inflammatory-related processes [24, 25]. Therefore, the improved inflammation in the BH group might be related to the low expression of ANGPTL-4. After BH treatment, DN-related biochemical indicators were returned to normal levels, to varying degrees. Urea, creatinine, triglycerides and total cholesterol levels were decreased, whereas the albumin level was increased compared with the DN group. Additionally, the ANGPTL-4 level positively correlated with the amount of 24-h urine protein, creatinine and the kidney weight index, and negatively correlated with total cholesterol according to Spearman analysis. Thus, we conclude that ANGPTL-4 might be associated with the regulation of these biochemical indicators by BH.

However, this study also has some limitations. A previous study showed that STZ could not only induce DN but also hypertension [26]. Because this study was focused on the effects of BH on DN, blood pressure was not measured and the influence of hypertension on ANGPTL-4 was not considered. Therefore, the effect of hypertension on ANGPTL-4 should be investigated in further studies. Despite this, the present study also demonstrated that BH can decrease proteinuria and improve DN, which might be associated with ANGPTL-4. In addition, this study revealed that ANGPTL-4 might be associated with the treatment effects of BH on DN. However, whether BH improved DN by targeting ANGPTL-4 is still unclear and a further study focused on the overexpression or deficiency of ANGPTL-4 should be performed to investigate a possible causal relationship between ANGPTL-4 expression and the effects of BH on improvement of DN.

## Conclusions

In conclusion, this study demonstrated that BH decreased proteinuria and improve DN, which might be closely associated with ANGPTL-4. However, the specific mechanism is not fully understood and needs to be examined in further studies.

## Abbreviations

ACEI: Angiotensin-converting enzyme inhibitor
BH: Benazepril hydrochloride
DBA: Diaminobenzidine
DN: Diabetic nephropathy
ESRD: End-stage renal disease
H&E: Haematoxylin and eosin
IDF: International Diabetes Federation
MCD: Minimal change disease
PBS: Phosphate-buffered saline
SD: Standard deviation
